# Analysis of Physician Compliance with Guideline-Directed Medical Therapy for Patients with Heart Failure with Reduced Ejection Fraction: A Real-World Study

**DOI:** 10.31083/j.rcm2409257

**Published:** 2023-09-18

**Authors:** Guixia Wang, Liming Liu, Xiaobo Wang, Ting Yu, Hui Xu, Tingjun Zhang, Jiafu Lin, Hao Luo, Yanxu Liu, Lanxiang Jiang, Wenlong Hu, Houxiang Hu

**Affiliations:** ^1^The First Affiliated Hospital, Jinan University, 510630 Guangzhou, Guangdong, China; ^2^Infectious Department, Affiliated Hospital of North Sichuan Medical College, 2262110 Nanchong, Sichuan, China; ^3^Department of Gerontology, Affiliated Hospital of North Sichuan Medical College, 2262110 Nanchong, Sichuan, China; ^4^Department of Cardiology, Affiliated Hospital of North Sichuan Medical College, 637002 Nanchong, Sichuan, China; ^5^Undergraduate in Clinical Medicine, North Sichuan Medical College, 2262110 Nanchong, Sichuan, China

**Keywords:** heart failure, guideline-directed medical therapy, compliance, real-world study

## Abstract

**Background:**

Although compliance with the guideline recommendations for heart failure (HF) is associated with improved survival, the effects of medication on clinical practice often fail to meet expectations due to physician and/or patient-related reasons that are unclear. This study analyzed physicians’ compliance with guideline-directed medical therapy (GDMT) based on real-world clinical data and identified risk factors of low compliance.

**Methods:**

This study included patients with HF, who were treated at the Affiliated Hospital of North Sichuan Medical College from July 2017 to June 2021. All patients were divided into high compliance, moderate compliance, and low compliance with GDMT groups. The proportion of patients receiving treatment in compliance with GDMT was analyzed, the relationship between compliance with GDMT and clinical outcomes was evaluated, and the risk factors of low compliance were identified.

**Results:**

Of all patients with HF included in the study, 498 (23.8%) had low compliance with GDMT, 1413 (67.4%) had moderate compliance with GDMT, and 185 (8.8%) had high compliance with GDMT. The readmission rate of patients in the moderate compliance with GDMT group was significantly higher than that in the high and low compliance groups (*p* = 0.028). There were no significant differences in the rates of severe cardiovascular disease among the three groups. The mortality rate of patients in the high compliance with GDMT group was significantly higher than that of the other groups (*p* < 0.001). We found that a history of hypertension; New York Heart Association (NYHA) classification (III and IV vs. I); and abnormal heart rate, high-sensitive troponin T (hsTnT), N-terminal prohormone of brain natriuretic peptide (NT-proBNP), uric acid, and left ventricular diastolic dysfunction (LVDD) were all significantly associated with low compliance with GDMT.

**Conclusions:**

The proportion of physicians’ compliance with GDMT in treating patients with HF is low. Risk factors of low compliance include hypertension; NYHA classification (III and IV vs. I); and abnormal heart rate, hsTnT, NT proBNP, uric acid, and LVDD.

## 1. Introduction

Chronic heart failure (HF) is a major public health problem worldwide, placing a significant burden on health systems [1]. HF is associated with high morbidity and mortality, with 50–75% of patients dying within 5 years of diagnosis [2]. Globally, the number of cases of end-stage HF is increasing at a rate of more than 800,000 per year, with a 1-year mortality rate of 70% and a sudden death rate of 60% [3]. A National Population-Based Analysis in China found that the age-standardized prevalence and incidence of HF are 1.10% and 275/100,000 person-years, respectively, and both prevalence and incidence increase with age [4]. Although treatment outcomes for chronic HF have improved with the development of new drugs and medical devices, HF is still associated with high rates of mortality and readmissions [5]. There are many potential reasons for this phenomenon, and non-compliance with guidelines is one of the important influencing factors.

Medication is a major component of HF treatment, which can not only relieve symptoms and prevent disease progression but also improve the quality of life and prolong the survival of HF patients. HF guidelines recommend the use of the maximum tolerated target dose of angiotensin-converting enzyme inhibitors (ACEIs) or angiotensin-receptor blockers (ARBs), beta blockers (BBs), mineralocorticoid receptor antagonists (MRAs), ivabradine, and angiotensin-receptor-neprilysin inhibitors (ARNIs) to reduce mortality and/or readmission rates due to HF [5, 6]. Several studies have also shown that the use of these drugs can reduce morbidity and mortality in patients with HF [7, 8]. One study found that better compliance with HF with reduced ejection fraction (HFrEF) guidelines is associated with better 60-day composite endpoints in HF with preserved EF (HFpEF) patients with atrial fibrillation [9]. Although compliance with the guideline recommendations for HF is associated with improved survival, the effects of medication in clinical practice often fail to meet expectations due to physicians and/or patient-related reasons that are unclear [10, 11, 12]. There is a persistent and observable gap in outpatient and inpatient HFrEF patients receiving guidance-directed medication (GDM) [13]. It takes a lot of time and work to implement the guideline recommendations into clinical practice.

Drug noncompliance is a major challenge for many chronic diseases with complex daily medication regiments. The Adherence to guideline-directed medical and device Therapy in outpAtients with heart failure with reduced ejection fraction (ATA) study showed that the majority of eligible HFrEF patients did not receive pharmacological therapy at the target dose or treatment with the device recommended by the guidelines [14]. Another study found that non-compliance with guidance-recommended medication in patients with HF is significantly associated with worsening symptoms, frequent hospitalizations, and premature death [15]. In addition, non-compliance with guidelines can lead to unnecessary treatments, tests, and invasive interventions that put patients at risk and waste significant financial costs [16, 17]. Therefore, improving compliance with guidelines is helpful for improving treatment outcomes, prognosis, and quality of life in patients with HF. There are many factors that affect guideline compliance, including patient-related factors (e.g., age, sex, financial income, disease awareness, education, comorbidities, disease severity) and physician-related factors (e.g., inadequate understanding of guidelines, safety concerns, patient’s personal reasons for dosing adjustment, doctor’s personal prescribing habits) [18, 19, 20].

At present, there is a lack of research analyzing the compliance of Chinese physicians with the treatment guidelines for HF and the risk factors. Based on real-world clinical data, this study analyzed physicians’ compliance with guidelines when treating patients with HF, as well as the impact on the clinical outcome of patients, and identified the risk factors of low compliance with GDM therapy (GDMT) from the patient’s perspective.

## 2. Methods

### 2.1 Patients

This was a real-world study involving patients with HF who were treated at the Affiliated Hospital of North Sichuan Medical College (Nanchong, China) from July 2017 to June 2021. Inclusion criteria included meeting the diagnostic and treatment standards for HFrEF in the “Chinese HF Diagnosis and Treatment Guidelines 2018”, follow-up for at least 6 months, receiving inpatient or outpatient care at the hospital, ≥18 years old with chronic HF, and diagnosed with left ventricular EF ≤40% (on the most recent echocardiogram, ≤2 years). The study excluded patients with follow-up less than half a year and patients with missing follow-up data, malignant tumors or other fatal diseases, had a history of major cardiac therapy such as heart transplantation or left ventricular assist device implantation, or had history of acute HF. All patients were divided into high compliance, moderate compliance, and low compliance with GDMT groups. The study analyzed the proportion of patients receiving treatment in compliance with GDMT, evaluated the relationship between compliance with GDMT and clinical outcomes, and identified the risk factors of low compliance. The study was approved by the Ethics Committee of the Affiliated Hospital of North Sichuan Medical College, and since the study only involved a retrospective analysis of previous clinical data, the requirement for informed consent was waived.

### 2.2 Variable Extraction

The data used in this study were extracted from a database constructed by combining information from multiple data sources including the Hospital Information System, Laboratory Information Management System, Picture archiving and Communication Systems, and Electronic Medical Record of the Affiliated Hospital of North Sichuan Medical College. The variables of interest in this study included patients’ sociodemographic information, drinking history, smoking history, previous medical history, comorbidities, HF-related characteristics, New York Heart Association (NYHA) functional class, laboratory parameters, medication, and other treatments.

### 2.3 Outcomes and Definition

Compliance with guideline score was based on physicians’ compliance with the latest European Society of Cardiology (ESC) HF guideline recommendations at the time the study registry was established [21]. Scores were related to the following five classes of medications recommended by the ESC: ACEI, ARB (if ACEI was not tolerated), ARNI, BB (it is recommended that all patients with HFrEF be prescribed an ACEI [or ARB or ARNI] and BB, except in cases of contraindications or intolerance), MRA (I. Patients with HFrEF NYHA class II–IV with EF ≤35% who remained symptomatic, although already on an ACEI [or ARB or ARNI] and BBs; II. All patients after an acute myocardial infarction who had an EF ≤40% with symptoms of HF or who had diabetes mellitus [DM]), and ivabradine (sinus rhythm, heart rate ≥70 bpm, NYHA class II–III, who were already receiving GDMT, including BBs at the maximum tolerated dose).

The compliance score was the ratio of treatment actually prescribed to what should theoretically have been prescribed. The treatment score was calculated for every drug for every patient prescribed, taking into account treatment eligibility criteria, guideline-based contraindications to drugs, side effects of the drug, and intolerance to the drug documented. The score was calculated for each patient by summing the points as follows: 0 points for non-prescription in the absence of contraindications and 1 point for the use of medicine; non-administration of the recommended drugs because of specific contraindications or intolerance was scored as compliance with guidelines. The total score ranged from 0 (very poor) to 1 (good) and we defined three levels of compliance: good compliance (score = 1), moderate compliance (score >0.5 to ≤0.75), and poor compliance (score ≤0.5). In this study, the term “compliance” only related to physicians’ compliance with guidelines and not to patients’ adherence or persistence.

### 2.4 Statistical Analyses

Continuous variables were checked for normality by using the Kolmogorov–Smirnov test. The normally distributed continuous variables are presented as the mean ± standard deviation, and the skewed distributed continuous variables are presented as the median and interquartile range (IQR). Categorical variables are displayed as the number and percentage. Comparisons of variables among the three groups were determined by one-way analysis of variance, the Kruskal-Wallis test, or the chi-square test. Factors with *p* < 0.05 in the comparison analysis were further analyzed using multivariate ordinal logistic regression to screen the independent influencing factors of physicians’ guideline compliance. All statistical analyses were performed using R software (version 4.2.0, Lucent Technologies, Murray Hill, NJ, USA), and the threshold of statistical significance was *p* < 0.05.

## 3. Results

### 3.1 Comparison of Baseline Characteristics of Low, Moderate, and High Compliance with GDMT Groups

A total of 2096 patients with HF were included in this study, with an average age of 69.5 ± 11.0 years, most of whom were male (58.7%). Among these patients, there were 185, 1413, and 498 patients in the high, moderate, and low compliance with GDMT groups, respectively. Comparisons among groups of the baseline characteristics showed significant differences in hypertension (*p* = 0.048), renal insufficiency (*p* < 0.001), and history of cardiac resynchronization therapy (CRT) (*p* < 0.001). There were no significant differences in the other variables among the three groups (Table 1).

**Table 1.** **Correlation of physicians’ guideline adherence with basic characteristics**.

Variables		Total (N = 2096)	Physicians’ guideline adherence			*p *value
			Low (N = 498)	Moderate (N = 1413)	High (N = 185)	
Demographic characteristics						
Age (years)		69.5 ± 11.0	68.8 ± 10.8	69.7 ± 11.1	70.4 ± 11.3	0.169
Sex						0.366
	Female	866 (41.3)	193 (38.8)	592 (41.9)	81 (43.8)	
	Male	1230 (58.7)	305 (61.2)	821 (58.1)	104 (56.2)	
Height (cm)		160.9 ± 7.9	161.2 ± 7.5	160.8 ± 8.0	160.2 ± 8.2	0.339
Weight (kg)		61.6 ± 11.2	61.7 ± 10.8	61.5 ± 11.1	61.7 ± 12.6	0.921
BMI (kg/$m^{2}$)		23.7 ± 3.5	23.7 ± 3.3	23.7 ± 3.6	23.9 ± 3.9	0.697
Employment						0.460
	No	176 (8.4)	37 (7.4)	126 (8.9)	13 (7.0)	
	Yes	1920 (91.6)	461 (92.6)	1287 (91.1)	172 (93.0)	
Education level						0.234
	Primary school or below	1377 (65.7)	311 (62.4)	936 (66.2)	130 (70.3)	
	Middle school	612 (29.2)	158 (31.7)	403 (28.5)	51 (27.6)	
	Junior college	46 (2.2)	15 (3.0)	30 (2.1)	1 (0.5)	
	Bachelor or above	61 (2.9)	14 (2.8)	44 (3.1)	3 (1.6)	
Medical insurance						0.212
	No	183 (8.7)	48 (9.6)	125 (8.8)	10 (5.4)	
	Yes	1913 (91.3)	450 (90.4)	1288 (91.2)	175 (94.6)	
Smoking		844 (40.3)	210 (42.2)	557 (39.4)	77 (41.6)	0.519
Drinking		651 (31.1)	157 (31.5)	440 (31.1)	54 (29.2)	0.836
Diseases history						
	MI	91 (4.3)	13 (2.6)	68 (4.8)	10 (5.4)	0.088
	Angina	78 (3.7)	17 (3.4)	50 (3.5)	11 (5.9)	0.244
	Arrhythmia	117 (5.6)	21 (4.2)	82 (5.8)	14 (7.6)	0.194
	VHD	31 (1.5)	5 (1.0)	24 (1.7)	2 (1.1)	0.487
	HCM	2 (0.1)	0 (0.0)	2 (0.1)	0 (0.0)	0.616
	DCM	16 (0.8)	3 (0.6)	11 (0.8)	2 (1.1)	0.810
	Pulmonary hypertension	2 (0.1)	0 (0.0)	2 (0.1)	0 (0.0)	0.616
	COPD	78 (3.7)	18 (3.6)	52 (3.7)	8 (4.3)	0.900
	Diabetes	558 (26.6)	119 (23.9)	378 (26.8)	61 (33.0)	0.057
	Hypertension	1201 (57.3)	267 (53.6)	816 (57.7)	118 (63.8)	0.048
	Renal insufficiency	30 (1.4)	3 (0.6)	17 (1.2)	10 (5.4)	<0.001
	Hyperlipidemia	32 (1.5)	6 (1.2)	20 (1.4)	6 (3.2)	0.130
	Myocarditis	4 (0.2)	1 (0.2)	2 (0.1)	1 (0.5)	0.504
	Sleep disorders	13 (0.6)	6 (1.2)	7 (0.5)	0 (0.0)	0.118
	Hyperuricemia	18 (0.9)	2 (0.4)	14 (1.0)	2 (1.1)	0.445
Thyroid function						0.528
	Normal	2063 (98.4)	488 (98.0)	1394 (98.7)	181 (97.8)	
	Hyperthyroidism	28 (1.3)	9 (1.8)	15 (1.1)	4 (2.2)	
	Hypothyroidism	5 (0.2)	1 (0.2)	4 (0.3)	0 (0.0)	
Family history						
	Hypertension	80 (3.8)	22 (4.4)	55 (3.9)	3 (1.6)	0.230
	Diabetes	22 (1.0)	6 (1.2)	13 (0.9)	3 (1.6)	0.629
	CHD	38 (1.8)	13 (2.6)	23 (1.6)	2 (1.1)	0.271
	Stroke	5 (0.2)	2 (0.4)	3 (0.2)	0 (0.0)	0.595
	Myocardiopathy	3 (0.1)	1 (0.2)	2 (0.1)	0 (0.0)	0.826
	MI	5 (0.2)	1 (0.2)	4 (0.3)	0 (0.0)	0.745
	Heart failure	58 (2.8)	19 (3.8)	37 (2.6)	2 (1.1)	0.128
Operation History						
	PCI	329 (15.7)	75 (15.1)	232 (16.4)	22 (11.9)	0.255
	CABG	18 (0.9)	3 (0.6)	15 (1.1)	0 (0.0)	0.263
	ICD	7 (0.3)	2 (0.4)	5 (0.4)	0 (0.0)	0.703
	CRT	3 (0.1)	1 (0.2)	1 (0.1)	1 (0.5)	0.262
NYHA classification						<0.001
	I	438 (20.9)	143 (28.7)	279 (19.7)	16 (8.6)	
	II	932 (44.5)	253 (50.8)	612 (43.3)	67 (36.2)	
	III	588 (28.1)	86 (17.3)	426 (30.1)	76 (41.1)	
	IV	138 (6.6)	16 (3.2)	96 (6.8)	26 (14.1)	

Note: BMI, body mass index; MI, myocardial infarction; VHD, valvular heart disease; HCM, hypertrophic cardiomyopathy; DCM, dilated cardiomyopathy; COPD, chronic obstructive pulmonary disease; CHD, coronary heart disease; PCI, percutaneous coronary intervention; CABG, coronary artery bypass grafting; ICD, implantable cardioverter-defibrillator; CRT, cardiac resynchronization therapy; NYHA, New York Heart Association.

### 3.2 Analysis of Compliance with GDMT in Patients with HF

**Fig. 1.**
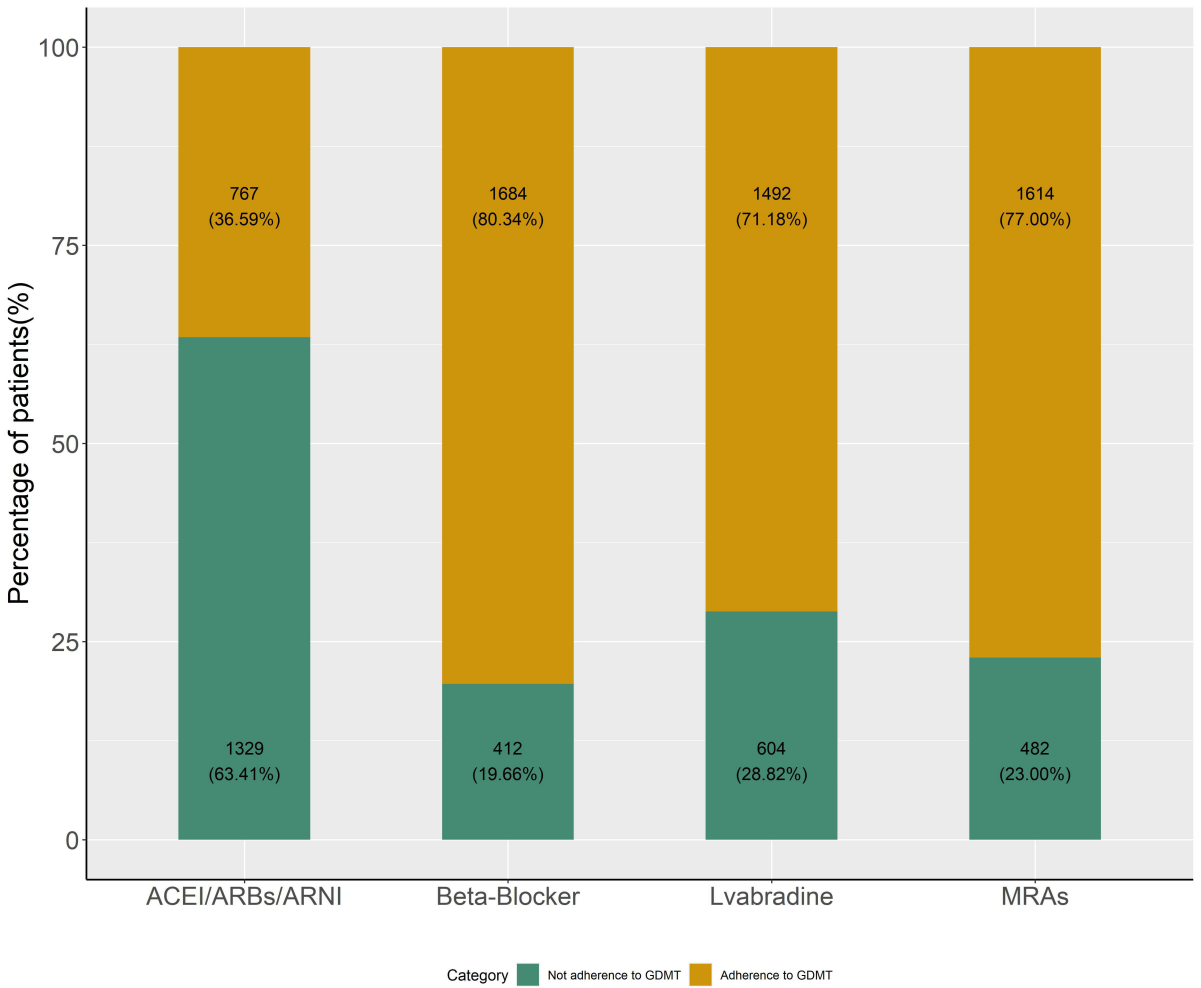
**Proportion of patients with HF receiving different drugs in compliance with GDMT**. HF, heart failure; GDMT, guideline-directed medical therapy; ACEI, angiotensin-converting enzyme inhibitor; ARBs, angiotensin-receptor blockers; ARNI, angiotensin-receptor-neprilysin inhibitor; MRAs, mineralocorticoid receptor antagonists.

**Fig. 2.**
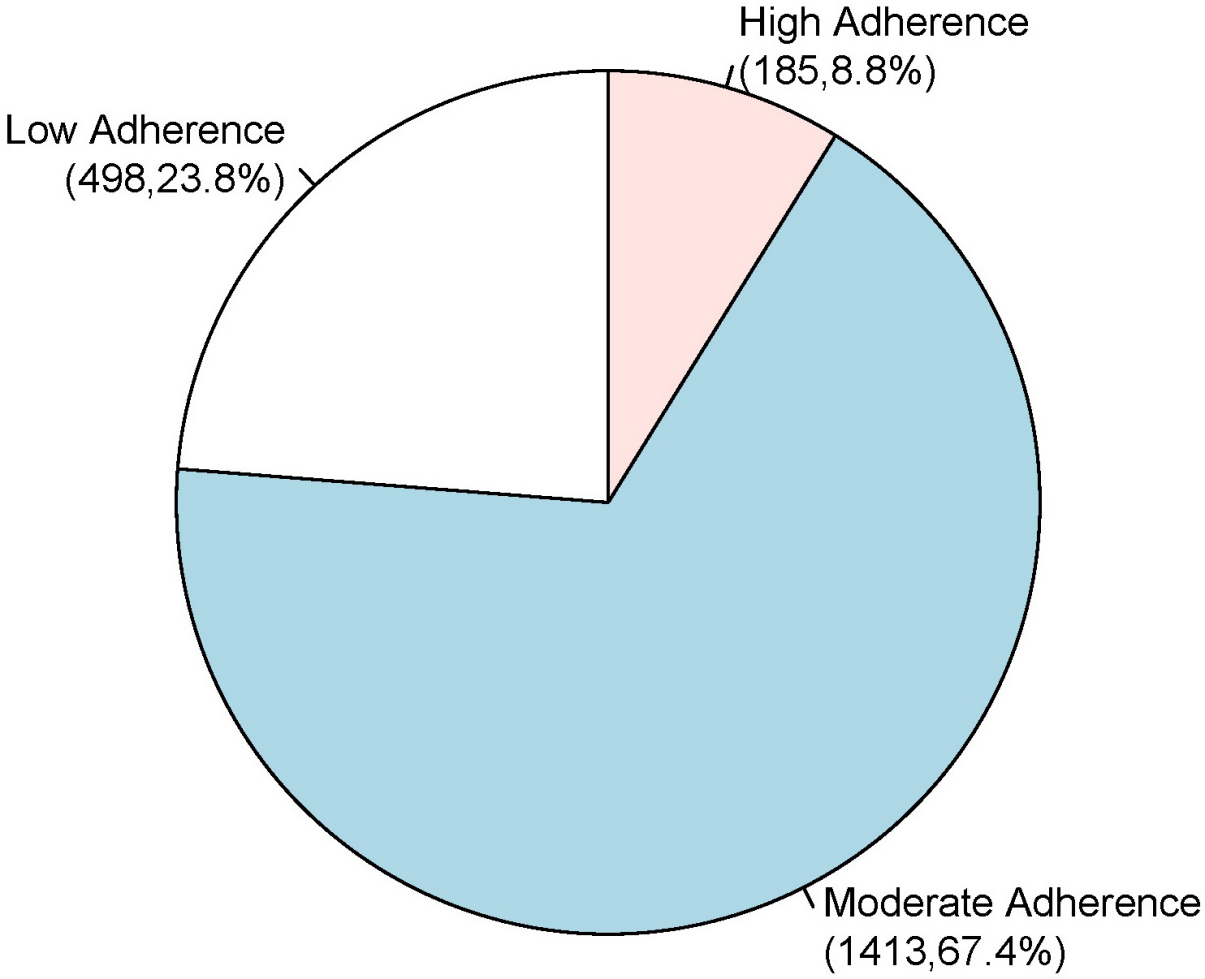
**Physicians’ guideline compliance**.

Our study found that 767 (36.59%) HF patients received ACEI/ARB/ARNI treatment, 1684 (80.34%) HF patients received beta blocker treatment, 1492 (71.18%) HF patients received ivabradine treatment, and 1614 (77.00%) HF patients received MRA treatment in compliance with GDMT (Fig. 1). Of all patients with HF included in the study, 498 (23.8%) had low compliance with GDMT, 1413 (67.4%) had moderate compliance with GDMT, and 185 (8.8%) had high compliance with GDMT (Fig. 2).

### 3.3 Analysis of Correlation between Clinical Outcomes and Physicians’ Guideline Compliance 

**Fig. 3.**
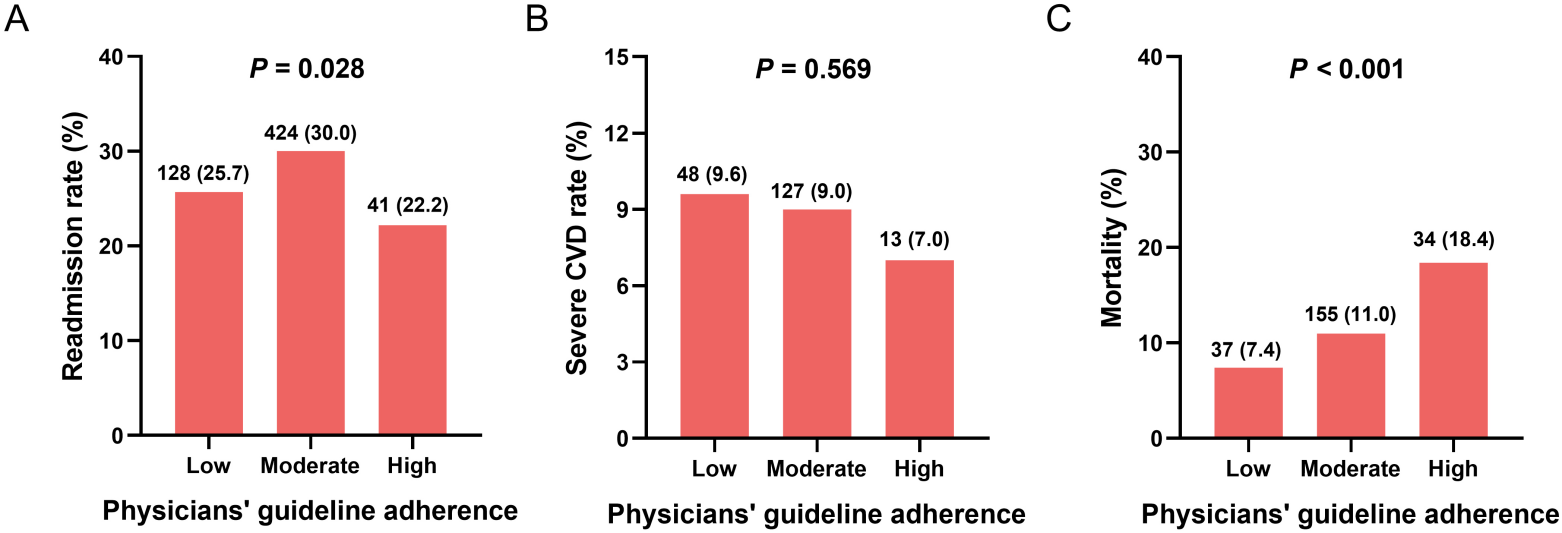
**Correlation of clinical outcomes with physicians’ guideline compliance**. (A) The correlation of readmission with physicians’ guideline adherence; (B) The correlation of severe CVD rate with physicians’ guideline adherence; (C) The correlation of mortality with physicians’ guideline adherence. CVD, cardiovascular disease.

The effect of compliance with treatment guidelines on clinical outcomes was analyzed. The readmission rate of patients in the moderate compliance with GDMT group was significantly higher than that in the high and low compliance with GDMT groups (*p* = 0.028; Fig. 3A). There were no significant differences in severe cardiovascular disease (CVD) rate among the low, moderate, and high compliance with GDMT groups (*p* = 0.569; Fig. 3B). Interestingly, patients in the high compliance with GDMT group had significantly higher mortality rates than those in the low and moderate compliance with GDMT groups (*p* < 0.001; Fig. 3C).

### 3.4 Comparison of Laboratory Indexes of Low, Moderate, and High Compliance with GDMT Groups

There were significant differences in heart rate (*p* < 0.001), diastolic blood pressure (DBP) (*p* = 0.030), systolic blood pressure (SBP) (*p* = 0.007), creatinine kinase MB (CK-MB) (*p* < 0.001), high-sensitive troponin T (hsTnT) (*p* < 0.001), N-terminal prohormone of brain natriuretic peptide (NT-proBNP) (*p* < 0.001), uric acid (*p* < 0.001), estimated glomerular filtration rate (eGFR) (*p* < 0.001), urea nitrogen (*p* < 0.001), serum creatinine (*p* < 0.001), alanine aminotransferase (ALT) (*p* = 0.046), aspartate aminotransferase (AST) (*p* < 0.001), alkaline phosphatase (ALP) (*p* = 0.028), gamma-glutamyl transpeptidase (GGT) (*p <* 0.001), total protein (*p* = 0.024), albumin (*p* < 0.001), prothrombin time (*p* = 0.001), fibrinogen (*p* = 0.011), red blood cells (*p* = 0.032), Free Triiodothyronine (FT3) (*p* < 0.001), Free Thyroxine (FT4) (*p* < 0.001), and left ventricular diastolic dysfunction (LVDD) (*p* < 0.001) among the three groups. There were no significant differences in other laboratory indexes among the three groups (Table 2).

**Table 2.** **Correlation of guideline adherence with laboratory index**.

Physical indexes		Total (N = 2096)	Physicians’ guideline adherence			*p *value
			Low (N = 498)	Moderate (N = 1413)	High (N = 185)	
Physical examination						
	Body temperature	36.5 (36.3–36.7)	36.5 (36.3–36.7)	36.5 (36.4–36.7)	36.5 (36.3–36.7)	0.186
	Heart rate	80.0 (73.0–95.0)	79.0 (68.0–80.0)	80.0 (75.0–99.0)	80.0 (74.5–102.0)	<0.001
	DBP	88.0 (82.0–95.0)	88.0 (82.0–93.0)	88.0 (82.0–96.0)	89.0 (82.0–98.0)	0.030
	SBP	145.0 (134.0–156.0)	145.0 (135.0–154.0)	145.0 (134.0–156.0)	150.0 (136.0–162.0)	0.007
Cardiac function indexes						
	CK-MB	1.960 (1.390–2.902)	1.875 (1.220–2.537)	1.960 (1.430–2.910)	2.340 (1.710–3.580)	<0.001
	hsTnT	0.021 (0.012–0.047)	0.016 (0.009–0.034)	0.021 (0.012–0.049)	0.033 (0.020–0.070)	<0.001
	NT-proBNP	773.3 (185.0–2259.2)	333.5 (90.4–1126.8)	786.8 (227.1–2329.0)	1688.0 (773.3–5569.0)	<0.001
Renal function indexes						
	Uric acid	371.7 (308.7–448.0)	359.8 (296.7–418.6)	371.7 (311.4–450.1)	418.9 (338.9–533.5)	<0.001
	eGFR	78.1 (62.5–94.0)	82.2 (67.9–97.6)	78.1 (62.7–92.8)	65.6 (40.8–84.6)	<0.001
	Urea nitrogen	6.100 (4.908–7.790)	5.760 (4.710–7.135)	6.100 (4.900–7.760)	7.320 (5.800–11.220)	<0.001
	Serum creatinine	76.9 (64.3–93.8)	74.1 (61.4–85.6)	76.9 (64.7–93.0)	90.7 (72.3–134.7)	<0.001
Liver function indexes						
	ALT	19.3 (13.7–28.9)	19.3 (13.0–26.0)	19.3 (14.0–29.3)	19.3 (13.3–31.0)	0.046
	AST	24.4 (19.9–32.0)	24.1 (18.8–28.4)	24.4 (20.1–33.3)	24.4 (20.6–33.2)	<0.001
	ALP	81.7 (69.0–95.5)	81.7 (67.0–92.8)	81.7 (70.0–96.1)	81.7 (69.9–97.7)	0.028
	GGT	30.5 (20.2–50.0)	30.0 (16.9–38.1)	30.5 (20.9–52.5)	31.0 (23.9–58.6)	<0.001
	Total protein	66.2 (63.1–69.5)	66.2 (63.4–69.1)	66.2 (63.2–69.7)	66.2 (61.5–68.3)	0.024
	Albumin	38.0 (35.6–40.3)	38.0 (36.4–40.6)	38.0 (35.5–40.2)	37.7 (34.1–39.6)	<0.001
	TBIL	13.6 (10.5–17.5)	13.6 (10.5–16.5)	13.6 (10.7–18.0)	13.6 (9.9–17.0)	0.139
	DBIL	3.400 (2.415–4.953)	3.400 (2.400–4.400)	3.400 (2.438–5.000)	3.400 (2.500–5.145)	0.207
	IBIL	10.0 (7.7–12.8)	10.0 (7.7–12.4)	10.0 (7.8–13.0)	10.0 (7.0–12.3)	0.077
Coagulation function						
	Prothrombin time	13.5 (12.8–14.4)	13.4 (12.7–14.1)	13.5 (12.9–14.5)	13.5 (13.0–14.8)	0.001
	APTT	35.5 (32.4–39.0)	35.5 (32.2–38.8)	35.5 (32.4–38.9)	35.5 (32.8–39.6)	0.647
	TT	17.2 (16.4–18.2)	17.2 (16.4–18.2)	17.2 (16.4–18.2)	17.2 (16.7–18.4)	0.328
	Fibrinogen	3.354 (2.882–3.970)	3.331 (2.812–3.828)	3.354 (2.900–4.010)	3.354 (2.982–4.200)	0.011
	Antithrombin III	89.4 (81.3–98.6)	89.6 (82.0–99.0)	89.4 (81.3–98.3)	89.4 (79.9–98.9)	0.169
Blood routine examination						
	WBC	6.300 (5.270–7.580)	6.300 (5.325–7.690)	6.300 (5.270–7.510)	6.300 (5.220–7.920)	0.392
	RBC	4.170 (3.800–4.530)	4.170 (3.850–4.570)	4.170 (3.810–4.520)	4.130 (3.550–4.550)	0.032
	HGB	132.0 (32.5–317.0)	132.0 (32.6–317.0)	132.0 (32.7–317.0)	128.0 (31.7–310.0)	0.136
	HCT	0.389 (0.350–0.422)	0.390 (0.354–0.427)	0.389 (0.350–0.421)	0.384 (0.331–0.420)	0.064
	Platelet	172.0 (135.0–210.0)	172.0 (138.0–215.0)	172.0 (133.0–209.0)	170.0 (131.0–207.0)	0.588
Thyroid function indexes						
	TSH	1.914 (1.358–2.788)	1.914 (1.529–2.780)	1.914 (1.293–2.825)	1.914 (1.225–2.727)	0.276
	FT3	2.685 (2.420–2.920)	2.685 (2.540–2.960)	2.685 (2.410–2.910)	2.685 (2.180–2.840)	<0.001
	FT4	1.270 (1.170–1.420)	1.270 (1.140–1.350)	1.270 (1.170–1.440)	1.270 (1.150–1.400)	<0.001
Echocardiography						
	LVDd	50.0 (45.00–55.00)	45.00(49.00–52.00)	45.0 (50.00–55.00)	50.0 (53.00–60.00)	<0.001
Electrocardiograph						
	Arrhythmia	117 (5.6)	21 (4.2)	82 (5.8)	14 (7.6)	0.194

Note: SBP, systolic blood pressure; DBP, diastolic blood pressure; CK-MB, creatine kinase-MB; hsTnT, high-sensitivity troponin T; NT-proBNP, N-terminal pro-B-type natriuretic peptide; eGFR, estimate glomerular filtration rate; ALT, alanine aminotransferase; AST, aspartate aminotransferase; ALP, alkaline phosphatase; GGT, gamma-glutamyl transpeptidase; TBIL, total bilirubin; DBIL, direct bilirubin; IBIL, indirect bilirubin; APTT, activated partial thromboplastin time; TT, thrombin time; WBC, white blood cell; RBC, red blood cell; HGB, hemoglobin; HCT, hematocrit; TSH, thyroid stimulating hormone; FT3, free triiodothyronine; FT4, free thyroxine; LVDd, left ventricular end-diastolic diameter.

### 3.5 Analysis of Factors Affecting Physicians’ Guideline Compliance Using Ordinal Logistic Regression

Ordinal logistic regression results showed that a history of hypertension (odds ratio [OR] = 1.332, 95% confidence interval [CI]: 1.090–1.628; *p = *0.005); NYHA classification (III vs. I) (OR = 1.569, 95% CI: 1.168–2.111; *p* = 0.003); NYHA classification (IV vs. I) (OR = 1.874, 95% CI: 1.180–2.974; *p* = 0.008); and abnormal heart rate (OR = 1.627, 95% CI: 1.312–2.021; *p* < 0.001), hsTnT (OR = 1.398, 95% CI: 1.104–1.771; *p* = 0.005), NT-proBNP (OR = 1.472, 95% CI: 1.150–1.886; *p* = 0.002), uric acid (OR = 1.398, 95% CI: 1.128–1.734; *p* = 0.002), and LVDD (OR = 1.358, 95% CI: 1.094–1.686; *p* = 0.006) were all significantly associated with low compliance with GDMT (Table 3).

**Table 3.** **Analysis of factors affecting physicians’ guideline adherence using ordinal logistic regression**.

Variables		β	SE	Wald χ^2^	*p* value	OR (95% CI)
History of MI		0.26	0.2342	1.1102	0.267	1.297 (0.821–2.055)
History of hypertension		0.286	0.1023	2.798	0.005	1.332 (1.090–1.628)
History of renal insufficiency		1.044	0.4151	2.5159	0.012	2.841 (1.238–6.329)
NYHA classification						
	I	Ref (1.000)				
	II	0.087	0.1233	0.705	0.481	1.091 (0.856–1.388)
	III	0.451	0.151	2.985	0.003	1.569 (1.168–2.111)
	IV	0.628	0.2358	2.663	0.008	1.874 (1.180–2.974)
Heart rate (abnormal)		0.487	0.1101	4.419	<0.001	1.627 (1.312–2.021)
DBP (abnormal)		0.136	0.1036	1.310	0.190	1.145 (0.935–1.404)
SBP (abnormal)		–0.028	0.1092	–0.258	0.797	0.972 (0.785–1.204)
CK-MB (abnormal)		0.024	0.1562	0.156	0.876	1.025 (0.755–1.393)
hsTnT (abnormal)		0.335	0.1205	2.783	0.005	1.398 (1.104–1.771)
NT-proBNP (abnormal)		0.387	0.1262	3.064	0.002	1.472 (1.150–1.886)
Uric acid (abnormal)		0.335	0.1096	3.055	0.002	1.398 (1.128–1.734)
eGFR (abnormal)		–0.11	0.1153	–0.950	0.342	0.896 (0.715–1.123)
Urea nitrogen (abnormal)		0.236	0.1227	1.925	0.054	1.267 (0.997–1.613)
Serum creatinine (abnormal)		0.136	0.1044	1.299	0.194	1.145 (0.934–1.406)
ALT (abnormal)		–0.024	0.1441	–0.165	0.869	0.977 (0.737–1.296)
AST (abnormal)		–0.028	0.1356	–0.204	0.838	0.973 (0.746–1.269)
ALP (abnormal)		0.024	0.1722	0.138	0.890	1.024 (0.731–1.437)
GGT (abnormal)		0.134	0.1167	1.146	0.252	1.143 (0.910–1.438)
Total protein (abnormal)		–0.104	0.1037	–1.004	0.315	0.901 (0.735–1.104)
Albumin (abnormal)		0.017	0.1131	0.147	0.883	1.017 (0.814–1.269)
Prothrombin time (abnormal)		–0.074	0.1102	–0.673	0.501	0.928 (0.748–1.153)
Fibrinogen (abnormal)		0.154	0.1113	1.382	0.167	1.166 (0.938–1.452)
RBC (abnormal)		–0.04	0.1069	–0.372	0.710	0.961 (0.779–1.185)
FT3 (abnormal)		0.09	0.1311	0.690	0.490	1.095 (0.847–1.416)
FT4 (abnormal)		0.277	0.1959	1.413	0.158	1.319 (0.899–1.938)
LVDd (abnormal)		0.306	0.1102	2.773	0.006	1.358 (1.094–1.686)
Threshold 1		0.072	0.1685	0.427	0.669	–
Threshold 2		3.9623	0.1994	19.870	<0.001	–

Note: MI, myocardial infarction; NYHA, New York Heart Association; DBP, diastolic blood pressure; SBP, systolic blood pressure; CK-MB, creatine kinase-MB; hsTnT, high-sensitivity troponin T; NT-proBNP, N-terminal pro-B-type natriuretic peptide; eGFR, estimate glomerular filtration rate; ALT, alanine aminotransferase; AST, aspartate aminotransferase; ALP, alkaline phosphatase; GGT, gamma-glutamyl transpeptidase; RBC, red blood cell; FT3, free triiodothyronine; FT4, free thyroxine; LVDd, left ventricular end-diastolic diameter.

### 3.6 Stratification Analysis of the Correlation of Clinical Outcomes with Physicians’ Guideline Compliance

This study conducted stratified analysis on patients with HF with different NYHA scores and compared the impact of guideline compliance on clinical outcomes in these patients. In patients with I/II NYHA, there were significant differences in readmission rates (*p* = 0.033) and mortality rates (*p* = 0.001) among the high, moderate, and low compliance with GDMT groups. There were no significant differences in severe CVD rates (*p* = 0.913) among the three groups. In patients with III/IV NYHA, there were no significant differences in readmission rates (*p* = 0.317), mortality rates (*p* = 0.766), and severe CVD rates (*p* = 0.725) among the three groups (Table 4). This study also compared the influence of basic characteristics and laboratory index of patients with I/II NYHA on physicians’ compliance with guidelines. The results are shown in **Supplementary Tables 1,2**.

**Table 4.** **Stratification analyzed the correlation of clinical outcomes with physicians’ guideline adherence**.

Variables			Physicians’ guideline adherence			*p *value
			Low	Moderate	High	
Patients with I/II NYHA (N = 1370)						
	Readmission					0.033
		No	302 (76.26)	631 (70.82)	67 (80.72)	
		Yes	94 (23.74)	260 (29.18)	16 (19.28)	
	Death					0.001
		No	377 (95.20)	823 (92.37)	69 (83.13)	
		Yes	19 (4.80)	68 (7.63)	14 (16.87)	
	Severe CVD					0.913
		No	357 (90.15)	803 (90.12)	76 (91.57)	
		Yes	39 (9.85)	88 (9.88)	7 (8.43)	
Patients with III/IV NYHA (N = 726)						
	Readmission					0.317
		No	68 (66.67)	358 (68.58)	77 (75.49)	
		Yes	34 (33.33)	164 (31.42)	25 (24.51)	
	Death					0.766
		No	84 (82.35)	435 (83.33)	82 (80.39)	
		Yes	18 (17.65)	87 (16.67)	20 (19.61)	
	Severe CVD					0.725
		No	93 (91.18)	483 (92.53)	96 (94.12)	
		Yes	9 (8.82)	39 (7.47)	6 (5.88)	

Note: NYHA, New York Heart Association; CVD, cardiovascular disease.

## 4. Discussion

Our study provides important real-world data on physician’s compliance with GDMT in patients with HFrEF in China and its impact on clinic outcomes. Our analysis showed that: (i) older age and comorbidities including hypertension, DM, and renal dysfunction were more common in Chinese HF patients with decreased EF; (ii) physicians’ compliance with GDMT and overall compliance score were good in 8.8%, moderate in 67.4%, and low in 23.8% of patients; and (iii) physicians’ high compliance was associated with better outcomes (reduction in CVD and HF rehospitalization) at the 24-month follow-up according to univariate analysis.

### 4.1 Population Profile

Our research describes in detail the clinical profile of our patients, who had a mean age of 69.5 years, tended to be older than those reported in previous studies, had an SBP of 145 mmHg, and had higher rates of common comorbidities including hypertension (57.3%) and DM (26.6%). Compared with the ASIAN-HF registry [22], device use was low in our study, with only 0.3% using an implantable cardioverter defibrillator and 0.1% undergoing CRT; this discrepancy may be partly related to socioeconomic considerations.

### 4.2 Guideline Compliance Level 

There is clear evidence from drug research studies showing that drugs recommended by the guidelines improve the clinical outcome of HF patients [23, 24]; however, there is still a large gap between GDMT and clinical practice [22, 25, 26]. We found that physicians’ compliance score was good in 8.8%, moderate in 67.4%, and low in 23.8% of patients, which was significantly lower than the QUALIFY global survey in which adherence was good in 67%, moderate in 25% and poor in 8% of patients [27]. Thus, in our study, there were still a large number of patients who received unoptimized treatment with just one or two of these medications, opposed to combination therapy, as recommended by guidelines, and only 8% of the patients received optimized treatment. Among eligible candidates with HFrEF, physicians tended to be in relative moderate compliance with BBs (80.3%) and MRAs (77%), similar to that of European countries; and in poor compliance with ACEI /ARB/ARNI (added up to 36.6%), which was much lower than that of European countries [12, 26, 28, 29] and the China PEACE Retrospective acute myocardial infarction (AMI) Study [30]. The high rate of use of MRAs in our study could be attributed to a nationwide quality assessment evaluation program [31] and the low cost of MRA. Poor compliance with ACEI was presumably due, in part, to the higher prevalence of persistent cough resulting from ACEI [32]. Contraindication to severe chronic kidney disease or renal failure also plays an important role. However, physicians’ awareness, including concern of adverse effects after combination therapy or during dose escalation for older patients, and economic factors also affecting physicians’ compliance level.

Although previous studies have encouraged uptitration, underdosing still remained significant in our study. A substantial proportion of patients with HFrEF received doses considerably below the guideline-recommended doses, especially for BBs. In view of this situation, the Chinese guidelines emphasize that BBs should gradually reach the target dose or maximum tolerance to facilitate clinical implementation, which lowers resting heart rate to 60 beats⋅$\min^{-1}$ [33]. Studies have proven that Asians achieve similar benefits as Westerners at lower statin doses [34, 35]. In addition to the low body mass index, complexities of the heterogeneous populations [36] and the heightened drug sensitivity of Asians, together with the pharmacokinetic variability [34, 35], may explain why prescribed doses were lower for patients in our study than western populations.

### 4.3 Relationship between Compliance and Clinical Outcomes

Data on the impact of physicians’ compliance with GDMT on clinical outcomes in daily practice are limited, particularly in China. We evaluated the physicians’ compliance with GDMT as well as the relationship between guideline compliance and clinical outcomes in hospitalized and outpatients with HFrEF in China for the first time. We found that high compliance with treatment guidelines was independently correlated with the low rate of HF or CVD rehospitalization, consistent with other studies [22, 37]. By contrast, we observed no significant benefit on mortality, which was inconsistent with another study [38]. Interestingly, we also found that physicians’ compliance was related to mortality in patients with NYHA I/II but not in patients with NYHA III/IV. To explore additional reasons, we conducted a subgroup analysis, which showed that in the group of patients with NYHA I/II, higher mortality was strongly correlated with comorbidities such as hypertension and renal insufficiency. The possibility that compliance had less impact on mortality than on hospitalization may be explained by the fact that mortality for HF is likely to be affected by several medical and non-medical factors including characteristics of the population baseline in our study, such as higher co-morbidities, older age, frailty, and poor financial situation. It is all the more interesting that aggressive treatment is often for patients in the more severe NYHA functional class in order to provide symptom relief. Thus, in our study, physicians’ compliance was better in patients with NYHA I/II with higher mortality risk but not in patients with NYHA III/IV. These findings are in accordance with the previously described “risk-treatment paradox”, where HF patients with the greatest need are less likely to receive appropriate therapy [39]. As quality improvement programs including improving the health care system and medical insurance systems, or a series of physician professional level training programs have been developed over the past years to improve the daily care of patients with HF in China, an increasing number of patients with HFrEF have been treated GDMT. With the increased demand to improve the quality of medical care in China, more efforts are needed to perform improvement measures and optimize the quality of data-based digital management systems, which have been shown to be efficient.

## 5. Limitations

Some important limitations of our study must be acknowledged. First, as this was a hospital-based, retrospective, observational study, it was limited by the nature of its design. We acknowledge that observational data cannot definitively establish causality or drug efficacy. Randomized controlled trials are required for definitive answers. Second, we performed multivariate regression analysis, but other unmeasured and hidden confounding variables such as patients’ compliance, whether patients take medication after discharge, socioeconomic factors, and health care system factors may have obvious impacts on clinical outcome [40]. Third, as with all studies that rely on automated sources of data, it is possible that parameters such as billing codes could be biased and proxies could fail to capture certain factors that are difficult to ascertain from the available clinical data. Finally, we did not analyze the relationship between dose and clinical results in our study, and the compliance score was measured only by the number of classes. We did not take dosage into account, as we only recorded the dosage of a given recommended class, and thus cannot provide a detailed explanation for underdosing or at which stage drug titration occurs.

## 6. Conclusions

In our real-word survey of inpatient and outpatient patients with HFrEF, we found that physicians’ compliance with HF class was not satisfactory, with just good in 8.8% of patient, and poor compliance with ACEI/ARB/ARNI. Furthermore, the underdosing of recommended medications was frequently observed, especially for BBs after discharge. This finding calls for action to improve combing drug pattern and uptitration of recommended therapies.

## Data Availability

Data is available from the corresponding author upon reasonable request.

## Supplementary Material

Supplementary material associated with this article can be found, in the online version, at https://doi.org/10.31083/j.rcm2409257.
