# Supplementary material for: Analysis of Physician Compliance with Guideline-Directed Medical Therapy for Patients with Heart Failure with Reduced Ejection Fraction: A Real-World Study
Source: Rev Cardiovasc Med. 2023 Sep 18;24(9):257. doi: 10.31083/j.rcm2409257 (PMC11270074; doi:10.31083/j.rcm2409257)
Supplement: Supplementary file 1 [file 2153-8174-24-9-257-s1.zip › 2153-8174-24-9-257-s1.docx]

Supplementary table 1. Correlation of physicians’ guideline adherence with basic characteristics in NYHA I/II patients

| Variables | Physicians’ guideline adherence | | | *P* value |
| --- | --- | --- | --- | --- |
|  | Low (N=396) | Moderate (N=891) | High (N=83) |  |
| Demographic characteristics |  |  |  |  |
| Age (years) | 68.4±10.8 | 68.6±11.0 | 68.9±12.2 | 0.926 |
| Sex |  |  |  | 0.593 |
| Female | 152 (38.4) | 364 (40.9) | 36 (43.4) |  |
| Male | 244 (61.6) | 527 (59.1) | 47 (56.6) |  |
| Height (cm) | 161.4±7.50 | 161.1±8.1 | 160.3±8.8 | 0.521 |
| Weight (kg) | 62.1±11.0 | 62.6±10.6 | 62.3±12.9 | 0.775 |
| BMI (kg/m^2^) | 23.8±3.2 | 24.1±3.3 | 24.2±3.9 | 0.341 |
| Employment |  |  |  | 0.723 |
| No | 30 (7.6) | 78 (8.8) | 8 (9.6) |  |
| Yes | 366 (92.4) | 813 (91.2) | 75 (90.4) |  |
| Education level |  |  |  | 0.241 |
| Primary school or below | 238(60.1) | 549(61.6) | 54(65.1) |  |
| Middle school | 133(33.6) | 282(31.6) | 28(33.7) |  |
| Junior college | 15(3.8) | 23(2.6) | 0(0.0) |  |
| Bachelor or above | 10(2.5) | 37(4.2) | 1(1.2) |  |
| Medical insurance |  |  |  | 0.212 |
| No | 360(90.9) | 819(91.9) | 79(95.2) |  |
| Yes | 36(9.1) | 72(8.1) | 4(4.8) |  |
| Smoking | 161(40.7) | 347(38.9) | 33(39.8) | 0.844 |
| Drinking | 126(31.8) | 272(30.5) | 26(31.3) | 0.896 |
| Diseases history |  |  |  |  |
| MI | 10(2.5) | 44(4.9) | 4(4.8) | 0.135 |
| Angina | 12(3.0) | 38(4.3) | 5(6.0) | 0.366 |
| Arrhythmia | 14(3.5) | 44(4.9) | 2(2.4) | 0.349 |
| VHD | 1(0.3) | 11(1.2) | 1(1.2) | 0.238 |
| HCM | 0(0.0) | 1(0.1) | 0(0.0) | 0.764 |
| DCM | 1(0.3) | 3(0.3) | 1(1.2) | 0.413 |
| COPD | 13(3.3) | 23(2.6) | 3(3.6) | 0.713 |
| Diabetes | 94(23.7) | 240(26.9) | 25(30.1) | 0.341 |
| Hypertension | 217(54.8) | 551(61.8) | 60(72.3) | **0.004** |
| Renal insufficiency | 2(0.5) | 6(0.7) | 4(4.8) | **<0.001** |
| Hyperlipidemia | 6(1.5) | 13(1.5) | 2(2.4) | 0.796 |
| Myocarditis | 1(0.3) | 0(0.0) | 0(0.0) | 0.292 |
| Sleep disorders | 5(1.3) | 4(0.4) | 0(0.0) | 0.186 |
| Hyperuricemia | 1(0.3) | 6(0.7) | 0(0.0) | 0.494 |
| Thyroid function |  |  |  | 0.229 |
| Normal | 390(98.5) | 884(99.2) | 81(97.6) |  |
| Hyperthyroidism | 6(1.5) | 5(0.6) | 2(2.4) |  |
| Hypothyroidism | 0(0) | 2(0.2) | 0(0) |  |
| Family history |  |  |  |  |
| Hypertension | 19(4.8) | 44(4.9) | 1(1.2) | 0.302 |
| Diabetes | 4(1.0) | 12(1.3) | 2(2.4) | 0.589 |
| CHD | 13(3.3) | 20(2.2) | 0(0.0) | 0.179 |
| Stroke | 1(0.3) | 3(0.3) | 0(0.0) | 0.850 |
| Myocardiopathy | 0(0.0) | 1(0.1) | 0(0.0) | 0.764 |
| MI | 1(0.3) | 4(0.4) | 0(0.0) | 0.735 |
| Heart failure | 17(4.3) | 29(3.3) | 0(0.0) | 0.137 |
| Operation History |  |  |  |  |
| PCI | 66(16.7) | 175(19.6) | 14(16.9) | 0.411 |
| CABG | 3(0.8) | 8(0.9) | 0(0.0) | 0.676 |
| ICD | 2(0.5) | 2(0.2) | 0(0.0) | 0.606 |
| CRT | 0(0.0) | 4(0.4) | 0(0.0) | 0.340 |

NOTE: BMI, Body mass index; MI, Myocardial infarction; VHD, Valvular heart disease; HCM, Hypertrophic cardiomyopathy; DCM, Dilated cardiomyopathy; COPD, Chronic obstructive pulmonary disease; CHD, Coronary heart disease; PCI, Percutaneous transluminal coronary intervention; CABG, Coronary artery bypass grafting; ICD, Implantable cardioverter defibrillator; CRT, Cardiac resynchronization therapy; NYHA, New York Heart Association; LVDd, Left ventricular end-diastolic diameter.

Supplementary table 2. Correlation of physicians’ guideline adherence with laboratory index in NYHA I/II patients

| Physical indexes | Physicians’ guideline adherence | | | *P* value |
| --- | --- | --- | --- | --- |
|  | Low (N=498) | Moderate (N=1413) | High (N=185) |  |
| **Physical examination** |  |  |  |  |
| Body temperature | 36.50 (36.30-36.70) | 36.50 (36.40-36.70) | 36.50 (36.35-36.70) | 0.516 |
| Heart rate | 77.50 (68.00-80.00) | 80.00 (72.00-89.00) | 80.00 (70.00-87.50) | **<0.001** |
| DBP | 88.00 (82.00-93.00) | 88.00 (82.00-96.00) | 88.00 (80.50-97.00) | **0.028** |
| SBP | 145.00 (135.00-154.00) | 145.00 (136.50-156.00) | 150.00 (138.50-162.00) | 0.050 |
| **Cardiac function indexes** |  |  |  |  |
| CK-MB | 1.73(1.15-2.40) | 1.89(1.34-2.71) | 1.96(1.44-3.21) | **0.001** |
| cTnT | 0.01(0.01-0.03) | 0.02(0.01-0.04) | 0.03(0.01-0.07) | **<0.001** |
| NT-proBNP | 212.65(70.71-773.30) | 519.00(135.05-1410.50) | 1084.00(403.70-3708.50) | **<0.001** |
| **Renal function indexes** |  |  |  |  |
| Uric acid | 350.45(288.28-407.85) | 364.60(306.40-426.28) | 400.30(337.42-512.22) | **<0.001** |
| eGFR | 85.28(72.34-99.50) | 79.22(67.00-97.54) | 71.91(54.09-87.84) | **<0.001** |
| Urea nitrogen | 5.61(4.60-6.76) | 5.86(4.78-7.34) | 6.74(5.71-9.04) | **<0.001** |
| Serum creatinine | 72.45(59.70-83.60) | 75.00(62.95-87.40) | 84.70(70.70-109.45) | **<0.001** |
| **Liver function indexes** |  |  |  |  |
| ALT | 19.30(12.67-25.58) | 19.30(13.95-27.00) | 19.30(13.50-28.95) | 0.246 |
| AST | 23.55(18.38-27.40) | 24.38(19.40-31.05) | 24.38(19.00-30.90) | **0.012** |
| ALP | 81.70(66.60-91.60) | 81.70(69.55-94.00) | 81.70(65.78-93.53) | 0.187 |
| GGT | 27.55(15.67-35.15) | 30.50(18.75-41.30) | 30.50(20.30-49.80) | **0.004** |
| Total protein | 66.25(63.30-69.10) | 66.25(63.52-69.80) | 65.60(61.78-66.95) | **0.012** |
| Albumin | 38.00(36.60-40.70) | 38.00(36.40-40.70) | 37.50(34.60-39.15) | **0.007** |
| TBIL | 13.60(10.10-16.07) | 13.60(10.20-16.40) | 13.60(9.70-16.85) | 0.721 |
| DBIL | 3.40(2.30-4.21) | 3.40(2.30-4.22) | 3.20(2.10-4.06) | 0.894 |
| IBIL | 10.00(7.57-12.30) | 10.00(7.60-12.30) | 10.00(7.20-11.85) | 0.708 |
| **Coagulation function** |  |  |  |  |
| Prothrombin time | 13.35(12.67-13.80) | 13.40(12.70-14.10) | 13.50(12.80-14.10) | 0.294 |
| APTT | 35.50(32.20-38.70) | 35.50(32.20-38.45) | 35.50(32.05-39.05) | 0.986 |
| TT | 17.20(16.40-18.10) | 17.20(16.40-18.15) | 17.20(16.40-18.10) | 0.889 |
| Fibrinogen | 3.35(2.82-3.83) | 3.35(2.91-3.98) | 3.36(3.05-4.43) | **0.016** |
| Antithrombin Ⅲ | 90.90(82.88-99.05) | 90.40(83.80-100.65) | 89.45(81.70-101.15) | 0.852 |
| **Blood routine examination** |  |  |  |  |
| WBC | 6.30(5.30-7.52) | 6.30(5.28-7.50) | 6.07(5.12-7.41) | 0.661 |
| RBC | 4.17(3.85-4.57) | 4.17(3.84-4.55) | 4.12(3.66-4.50) | 0.172 |
| HGB | 132.00(32.58-315.25) | 132.00(32.55-317.00) | 123.00(31.50-314.50) | 0.183 |
| HCT | 0.39(0.35-0.42) | 0.39(0.36-0.42) | 0.38(0.34-0.41) | 0.110 |
| Platelet | 172.00(137.75-217.00) | 172.00(137.00-213.00) | 166.00(130.00-219.00) | 0.942 |
| **Thyroid function indexes** |  |  |  |  |
| TSH | 1.91(1.53-2.72) | 1.91(1.28-2.73) | 1.91(1.18-2.80) | 0.284 |
| FT3 | 2.70(2.60-3.00) | 2.68(2.53-2.96) | 2.68(2.22-2.92) | **0.029** |
| FT4 | 1.27(1.13-1.34) | 1.27(1.16-1.40) | 1.27(1.13-1.38) | **0.018** |
| **Echocardiography** |  |  |  |  |
| LVDd | 48.00(44.00-51.00) | 49.00(45.00-52.00) | 51.00(48.00-57.00) | **<0.001** |
| **Electrocardiograph** |  |  |  |  |
| Arrhythmia | 14(3.5) | 44 (4.9) | 2 (2.4) | 0.349 |

NOTE: SBP, Systolic blood pressure; DBP, Diastolic blood pressure; CK-MB, creatine kinase-MB; hsTnT, High-sensitivity troponin T; NT-proBNP, N-terminal pro-B-type natriuretic peptide; eGFR, Estimate Glomerular Filtration Rate; ALT, Alanine aminotransferase; AST, Aspartate aminotransferase; ALP, Alkaline phosphatase; GGT, Gamma-glutamyl transpeptidase; TBIL, Total bilirubin; DBIL, Direct Bilirubin; IBIL, Indirect bilirubin; APTT, Activated Partial Thromboplastin Time; TT, thrombin time; WBC, White blood cell; RBC, Red blood cell; HGB, hemoglobin; HCT, hematocrit; TSH, Thyroid stimulating hormone; FT3, Free triiodothyronine; FT4, Free thyroxine; LVDd, Left ventricular end-diastolic diameter.
